# Obstetric interventions and pregnancy outcomes during the COVID-19 pandemic in England: A nationwide cohort study

**DOI:** 10.1371/journal.pmed.1003884

**Published:** 2022-01-10

**Authors:** Ipek Gurol-Urganci, Lara Waite, Kirstin Webster, Jennifer Jardine, Fran Carroll, George Dunn, Alissa Frémeaux, Tina Harris, Jane Hawdon, Patrick Muller, Jan van der Meulen, Asma Khalil

**Affiliations:** 1 Royal College of Obstetricians and Gynaecologists, London, United Kingdom; 2 Department of Health Services Research, Faculty of Public Health and Policy, London School of Hygiene &Tropical Medicine, London, United Kingdom; 3 Faculty of Health and Life Sciences, De Montfort University, Leicester, United Kingdom; 4 Royal Free London NHS Foundation Trust, London, United Kingdom; 5 Fetal Medicine Unit, St George’s Hospital, London, United Kingdom; 6 Vascular Biology Research Centre, Molecular and Clinical Sciences Research Institute, St George’s, University of London, London, United Kingdom; 7 Fetal Medicine Unit, Liverpool Women’s Hospital, Liverpool, United Kingdom; Cambridge University, UNITED KINGDOM

## Abstract

**Background:**

The COVID-19 pandemic has disrupted maternity services worldwide and imposed restrictions on societal behaviours. This national study aimed to compare obstetric intervention and pregnancy outcome rates in England during the pandemic and corresponding pre-pandemic calendar periods, and to assess whether differences in these rates varied according to ethnic and socioeconomic background.

**Methods and findings:**

We conducted a national study of singleton births in English National Health Service hospitals. We compared births during the COVID-19 pandemic period (23 March 2020 to 22 February 2021) with births during the corresponding calendar period 1 year earlier. The Hospital Episode Statistics database provided administrative hospital data about maternal characteristics, obstetric inventions (induction of labour, elective or emergency cesarean section, and instrumental birth), and outcomes (stillbirth, preterm birth, small for gestational age [SGA; birthweight < 10th centile], prolonged maternal length of stay (≥3 days), and maternal 42-day readmission). Multi-level logistic regression models were used to compare intervention and outcome rates between the corresponding pre-pandemic and pandemic calendar periods and to test for interactions between pandemic period and ethnic and socioeconomic background. All models were adjusted for maternal characteristics including age, obstetric history, comorbidities, and COVID-19 status at birth. The study included 948,020 singleton births (maternal characteristics: median age 30 years, 41.6% primiparous, 8.3% with gestational diabetes, 2.4% with preeclampsia, and 1.6% with pre-existing diabetes or hypertension); 451,727 births occurred during the defined pandemic period. Maternal characteristics were similar in the pre-pandemic and pandemic periods. Compared to the pre-pandemic period, stillbirth rates remained similar (0.36% pandemic versus 0.37% pre-pandemic, *p* = 0.16). Preterm birth and SGA birth rates were slightly lower during the pandemic (6.0% versus 6.1% for preterm births, adjusted odds ratio [aOR] 0.96, 95% CI 0.94–0.97; 5.6% versus 5.8% for SGA births, aOR 0.95, 95% CI 0.93–0.96; both *p <* 0.001). Slightly higher rates of obstetric intervention were observed during the pandemic (40.4% versus 39.1% for induction of labour, aOR 1.04, 95% CI 1.03–1.05; 13.9% versus 12.9% for elective cesarean section, aOR 1.13, 95% CI 1.11–1.14; 18.4% versus 17.0% for emergency cesarean section, aOR 1.07, 95% CI 1.06–1.08; all *p <* 0.001). Lower rates of prolonged maternal length of stay (16.7% versus 20.2%, aOR 0.77, 95% CI 0.76–0.78, *p <* 0.001) and maternal readmission (3.0% versus 3.3%, aOR 0.88, 95% CI 0.86–0.90, *p <* 0.001) were observed during the pandemic period. There was some evidence that differences in the rates of preterm birth, emergency cesarean section, and unassisted vaginal birth varied according to the mother’s ethnic background but not according to her socioeconomic background. A key limitation is that multiple comparisons were made, increasing the chance of false-positive results.

**Conclusions:**

In this study, we found very small decreases in preterm birth and SGA birth rates and very small increases in induction of labour and elective and emergency cesarean section during the COVID-19 pandemic, with some evidence of a slightly different pattern of results in women from ethnic minority backgrounds. These changes in obstetric intervention rates and pregnancy outcomes may be linked to women’s behaviour, environmental exposure, changes in maternity practice, or reduced staffing levels.

## Abstract 2

In a nationwide cohort study, Ipek Gurol-Urganci and colleagues study obstetric intervention rates and pregnancy outcomes in England during the first year of the COVID-19 pandemic.

## Author summary

**Why was this study done?:**

- The COVID-19 pandemic has been associated with a reduction in the rates of preterm births and small-for-gestational-age births in some high-income settings.
- It is unknown how changes in high-income settings have influenced rates of obstetric interventions, and whether the pandemic has exacerbated existing inequalities in maternity care and outcomes.

**What did the researchers do and find?:**

- We conducted a national population-based study, using administrative hospital records from England to compare obstetric intervention rates and maternal outcomes during the pandemic period and the corresponding calendar period 1 year earlier.
- Rates of obstetric interventions were slightly higher in England during the pandemic than in the pre-pandemic period.
- There was some evidence of slightly lower rates of preterm birth and small-for-gestational-age birth.
- There were very small variations in the pattern of results according to women’s ethnic background.

**What do these findings mean?:**

- The COVID-19 pandemic was not associated with overall worse pregnancy outcomes in England. However, there was a higher rate of obstetric interventions, which may have been the consequence of lockdown restrictions and service interruptions.
- The results should be interpreted with caution. All differences were small, and many comparisons were made.

## Introduction

During the COVID-19 pandemic, pregnant women are vulnerable to both the ‘direct effects’ of infection with SARS-CoV-2 and the ‘indirect effects’ of disruption of essential healthcare services and restrictions on social interaction. There is no evidence that pregnant women face an increased risk of contracting SARS-CoV-2 infection compared to non-pregnant women. However, there is evidence that women who experience a SARS-CoV-2 infection during pregnancy may have higher rates of maternal morbidity and mortality including preterm birth, preeclampsia, and emergency cesarean delivery; neonatal morbidity; and perinatal morbidity and mortality including stillbirth [1–4]. The most recent data from the UK Obstetric Surveillance System give a preterm birth rate of 21% and a cesarean birth rate of 43% for pregnant women who require admission to hospital with a SARS-CoV-2 infection [5].

There are reports that the impact of COVID-19 differs among ethnic and socioeconomic groups, with pregnant women from ethnic minority backgrounds more likely to be hospitalised [5]. Furthermore, the direct impact of the SARS-CoV-2 infection has been disproportionately high in ethnic minority groups and socioeconomically deprived communities [6].

The population-level impacts of the indirect effects of the pandemic on maternal and neonatal outcomes are, however, likely to be larger than direct effects of COVID-19 infection [7–9]. A recent systematic review and meta-analysis focusing on the indirect effects of the pandemic on maternity outcomes found that in low- and middle-income settings, the pandemic period has been associated with an increase in maternal mortality and stillbirths [7]. However, to the best of our knowledge, there is no evidence of such an association in high-income settings, and uncertainty remains about the broader impact of the pandemic.

Almost all available reports, with the exception of national data from the US, are restricted to the first 8 months of 2020 [7,10]. Consequently, there is little evidence regarding the indirect effects of the pandemic during the later months of 2020, when many women would have experienced changes to care and restrictions on social interaction for most or all of their pregnancy.

In this study, we used population-level data from England to examine the combined direct and indirect effects of the COVID-10 pandemic on mode of delivery and pregnancy outcomes. We compared obstetric intervention and pregnancy outcome rates in England during a defined ‘pandemic period’ (23 March 2020 to 22 February 2021, including 2 periods of national ‘lockdown’) with those during the corresponding pre-pandemic calendar period 1 year earlier (23 March 2019 to 22 February 2020) and tested whether differences in these rates varied according to ethnic and socioeconomic background.

During the pandemic period, there were major changes to healthcare delivery throughout the year [11]. For maternity care, there has been consistent national provision of rapid guidance and support from professional organisations to inform changes in service [12]. Changes were broadly similar to those seen across Europe, including increased use of remote consultation, reduced provision of out-of-hospital care and birth, and widespread restrictions on access for support partners [13].

## Methods

### Study design

This study is a national population-based study using Hospital Episode Statistics (HES) records of all inpatient admissions to National Health Service (NHS) hospitals in England [14]. HES records contain data on patient demographics (age, sex, and ethnicity), admission and discharge dates, and diagnostic and procedure information. Diagnostic information is coded using the International Classification of Diseases–10th revision (ICD-10) [15]. Procedures are coded using the UK Office for Population Censuses and Surveys Classification of Interventions and Procedures–4th revision (OPCS-4) [16]. Each episode related to the birth of a baby contains details of the labour and birth (e.g., gestational age and birthweight) in supplementary data fields known as the HES ‘maternity tail’.

### Cohort selection and outcome definitions

The pandemic period and the lockdown periods were defined according to the timeline of events related to the COVID-19 pandemic in England: the start of the first national lockdown (23 March 2020), the relaxation of lockdown restrictions and move to a tiered system of local restrictions (24 June 2020), the start of national measures leading to a second national lockdown (22 September 2020), and the relaxation of national lockdown restrictions (23 February 2021). We chose to handle time in this way because during the lockdown periods, there were substantial changes to care and staffing: Staff were redeployed away from maternity settings, and hospital access restrictions changed [17]. Further details are provided in S1 Table.

The cohort included all women with a record in HES of a singleton birth during the pandemic period (23 March 2020 to 22 February 2021) and the pre-pandemic period (the corresponding calendar period 1 year earlier: 23 March 2019 to 22 February 2020). A birth (maternity) episode was defined as any record that contained information about mode of birth in procedure fields (OPCS-4 codes: R17.1 to R25.9), the main record, or the maternity tail. Multiple births were excluded. Multiple births were defined as birth episodes with an ICD-10 code for a multiple birth (Z37.2–7) or strong evidence of a multiple birth in the maternity tail (more than 1 distinct birthweight, birth order, and baby recorded in the same birth episode).

The study outcomes included stillbirth (fetal death at ≥24 weeks’ gestation), preterm birth (less than 37 weeks’ gestation), small for gestational age (SGA) at birth (defined as birthweight < 10th centile using population-based centile charts [18]), induction of labour, mode of birth (instrumental vaginal birth, elective cesarean section, emergency cesarean section, or unassisted vaginal birth), prolonged maternal length of stay (3 or more days after birth), and maternal readmission within 42 days of birth. For maternal readmissions, due to the 6-week follow-up required to assess the outcome, the cohort is restricted to births up to 17 February 2021 as the most recent HES dataset available at the time of analysis included records of hospital episodes until 31 March 2021.

Maternal age was grouped into 5-year periods, with women under 20 and over 39 years being aggregated into single categories. Parity and previous cesarean section were determined using women’s previous HES hospital admission records. Ethnic background was coded using the Office for National Statistics (ONS) 2001 census categorisation for ethnicity, collapsed into 4 groups: White, South Asian, Black, and Other (combined Mixed and Other, including Chinese), as there is evidence of misclassification if more granular groups are used [19,20].

Information about comorbidities—including pre-existing and gestational diabetes mellitus, pre-existing hypertension, and preeclampsia—was available in the diagnosis codes in the HES records of the birth episode, with women assumed not to have these conditions if the relevant ICD-10 codes were not present. A woman was classified as having laboratory-confirmed SARS-CoV-2 infection at the time of birth if the ICD-10 code for ‘COVID-19, virus identified’ (U07.1) was recorded in the birth episode [21]. The test used to confirm infection in NHS hospital admissions is a nasal or throat swab examined using PCR [22].

The Index of Multiple Deprivation (IMD) was used as an overall measure of area-based deprivation. It is based on the income, education, employment, crime, and living environment in an individual’s area of residence [23]. Quintiles of the national distribution of 2019 IMD rankings of 32,844 Lower Super Output Areas, each with typically 1,500 inhabitants, were used to categorise women into 5 groups according to socioeconomic background. Definitions of maternal characteristics and study outcomes, and their coding and completeness in HES, are outlined in S2 Table.

### Statistical methods

All outcomes and analyses described in the Methods were prespecified, with the exception of the use of multiple imputation methods for missing data and case-mix adjustment. These modifications were done following editorial and peer review. We did not publish or pre-register an analysis plan. This study is reported as per the REporting of studies Conducted using Observational Routinely-collected Data (RECORD) guideline (S1 Text).

The characteristics of the women in the cohort were described for each analysis period, and monthly rates of different maternal and neonatal outcomes for the entire pre-pandemic and pandemic periods were plotted. Multi-level logistic regression models were used to estimate the differences in obstetric intervention and pregnancy outcome rates between the pandemic and the corresponding pre-pandemic calendar period in terms of odds ratios (ORs) and their 95% confidence intervals (CIs). ORs larger than 1 indicate that rates are increased during the pandemic period compared to the corresponding calendar period 1 year earlier. All analyses were adjusted for maternal age, obstetric history, comorbidities (pre-existing and gestational diabetes mellitus, pre-existing hypertension, and preeclampsia), and COVID-19 status at birth, and allowed for hospital-level clustering.

A binary variable for ethnicity (white versus all other categories, hereafter referred to as ‘ethnic minority backgrounds’) or socioeconomic background (‘less deprived’, defined as IMD quintiles 1 to 3, versus ‘more deprived’, defined as IMD quintiles 4 and 5) was included, and tests for interaction were used to determine if differences in outcome rates between pandemic and corresponding pre-pandemic periods varied according to ethnic and socioeconomic background. Missing values for maternal characteristics and outcomes were imputed using chained equations to generate 10 datasets; estimates from these datasets were pooled using Rubin’s rules [24]. A *p-*value of less than 0.05 was assumed to represent statistical significance. Stata 16 (StataCorp, College Station, TX) was used for all analyses.

### Ethical approval

This study used routinely collected administrative hospital data that were accessed to evaluate service provision and performance, and was therefore exempt from ethical review by the NHS Health Research Authority. The use of personal data without individual consent was approved by the NHS Health Research Authority (16/CAG/0058).

### Patient and public involvement

Neither women giving birth nor their representative organisations were explicitly consulted regarding this study. However, the outcome measures used were developed in collaboration with the National Maternity and Perinatal Audit (NMPA) Women and Families Group, and we will act in conjunction with this group to disseminate the findings of this study.

## Results

We identified 961,506 births in the English NHS during the pandemic period (23 March 2020 to 22 February 2021) and pre-pandemic period (23 March 2019 to 22 February 2020); 948,020 (98.6%) were singleton births. There was an overall 8.4% reduction in singleton births, from 493,293 in the pre-pandemic period to 451,727 in the pandemic period (S1 Fig). The characteristics of the women included were broadly similar in the pre-pandemic and pandemic periods (Table 1).

**Table 1:** Characteristics of pregnant women included in the study.

Characteristic	Pre-pandemic	Pandemic	Pandemic periods		
			COVID-19 1st lockdown	COVID-19 local restrictions	COVID-19 2nd lockdown
**Time period covered**	23 Mar 2019–22 Feb 2020	23 Mar 2020–22 Feb 2021	23 Mar 2020–23 Jun 2020	24 Jun 2020–21 Sep 2020	22 Sep 2020–22 Feb 2021
**Number of births**	496,293	451,727	126,093	124,171	201,463
**Age (years)** *					
≤19	14,361 (2.9)	12,005 (2.7)	3,431 (2.7)	3,418 (2.8)	5,156 (2.6)
20–24	69,020 (13.9)	59,756 (13.2)	16,781 (13.3)	16,639 (13.4)	26,336 (13.1)
25–29	138,118 (27.8)	123,572 (27.4)	34,644 (27.5)	34,362 (27.7)	54,566 (27.1)
30–34	162,839 (32.8)	152,578 (33.8)	42,117 (33.4)	41,664 (33.6)	68,797 (34.1)
35–39	91,400 (18.4)	84,141 (18.6)	23,674 (18.8)	22,669 (18.3)	37,798 (18.8)
40+	20,550 (4.1)	19,671 (4.4)	5,444 (4.3)	5,419 (4.4)	8,808 (4.4)
**Obstetric history**					
Primiparous	202,387 (40.8)	192,308 (42.6)	51,927 (41.2)	51,983 (41.9)	88,398 (43.9)
Multiparous with no previous CS	233,736 (47.1)	207,275 (45.9)	58,525 (46.4)	57,157 (46.0)	91,593 (45.5)
Multiparous with previous CS	60,170 (12.1)	52,144 (11.5)	15,641 (12.4)	15,031 (12.1)	21,472 (10.7)
**Comorbidities**					
Pre-existing diabetes	4,612 (0.9)	4,211 (0.9)	1,161 (0.9)	1,143 (0.9)	1,907 (0.9)
Gestational diabetes	40,177 (8.1)	39,215 (8.7)	10,304 (8.2)	10,649 (8.6)	18,262 (9.1)
Pre-existing hypertension	3,497 (0.7)	3,384 (0.8)	889 (0.7)	957 (0.8)	1,538 (0.8)
Preeclampsia or eclampsia	11,285 (2.3)	11,396 (2.5)	3,071 (2.4)	3,040 (2.5)	5,285 (2.6)
COVID-19-positive at birth	0 (0)	4,578 (1.0)	564 (0.4)	199 (0.2)	3,815 (1.9)
**Ethnicity** *					
White	336,484 (76.7)	307,799 (76.4)	85,096 (76.2)	83,934 (76.3)	138,769 (76.6)
South Asian	52,056 (11.9)	49,423 (12.3)	13,798 (12.4)	13,448 (12.2)	22,177 (12.2)
Black	20,517 (4.7)	18,344 (4.6)	5,190 (4.6)	5,085 (4.6)	8,059 (4.4)
Other	29,365 (6.7)	27,459 (6.8)	7,607 (6.8)	7,608 (6.9)	12,244 (6.8)
**Socioeconomic deprivation** *					
Quintile 1 = least deprived	73,691 (15.0)	68,480 (15.2)	19,003 (15.2)	18,495 (15.5)	30,982 (15.5)
Quintile 2	83,614 (17.0)	77,359 (17.2)	21,498 (17.1)	21,045 (17.0)	34,816 (17.4)
Quintile 3	94,921 (19.3)	86,637 (19.3)	24,124 (19.2)	23,820 (19.3)	38,693 (19.3)
Quintile 4	109,876 (22.3)	99,831 (22.2)	27,863 (22.2)	27,476 (22.3)	44,492 (22.2)
Quintile 5 = most deprived	130,446 (26.5)	116,874 (26)	32,900 (26.2)	32,614 (26.4)	51,270 (25.6)

Data are given as *n* (percent) unless otherwise indicated. CS, cesarean section.

*In the combined pre-pandemic and pandemic cohort, 9 (0.001%) records were missing information on maternal age, 106,583 (11.6%) records were missing information on ethnicity, and 6,381 (0.7%) records were missing information on Index of Multiple Deprivation.

**Fig 1:**
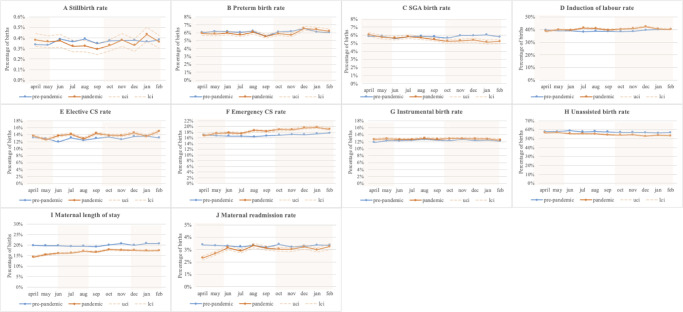
Study outcome rates by month in the pre-pandemic (April 2019–February 2020) and pandemic (April 2020–February 2021) periods. (A) Stillbirth; (B) preterm birth; (C) small for gestational age (SGA); (D) induction of labour; (E) elective cesarean section (CS); (F) emergency CS; (G) instrumental birth; (H) unassisted birth; (I) prolonged maternal le ngth of stay; (J) maternal readmission. lci, 95% lower confidence interval; uci, 95% upper confidence interval.

The monthly stillbirth rates are presented in Fig 1. Compared with the corresponding pre-pandemic calendar periods (Table 2), there were no statistically significant differences in stillbirth rates during the entire pandemic period (0.37% versus 0.36% pre-pandemic, adjusted OR [aOR] 0.95, 95% CI 0.89 to 1.02, *p =* 0.16), the first lockdown period (0.38% versus 0.35% in the same pre-pandemic calendar period, aOR 1.08, 95% CI 0.95 to 1.23, *p =* 0.24), or the second lockdown period (0.37% versus 0.38% in the same pre-pandemic calendar period, aOR 0.93, 95% CI 0.84 to 1.03, *p =* 0.18).

**Table 2:** Comparisons of outcomes in the COVID-19 pandemic and lockdown periods with the same periods in the previous year.

Outcome	Measure	Pandemic versus pre-pandemic period(time period covered: 23 March–22 February)				COVID-19 first lockdown versus previous year (time period covered: 23 March–23 June)				COVID-19 second lockdown versus previous year (time period covered: 22 September–22 February)			
		Pre-pandemic	Pandemic	OR (95% CI) *p*-value	aOR* (95% CI) *p-*value	Pre-pandemic	Pandemic	OR (95% CI) *p-*value	aOR* (95% CI) *p-*value	Pre-pandemic	Pandemic	OR (95% CI) *p-*value	aOR* (95% CI) *p-*value
Number of observations		493,293	451,727			136,161	126,093			224,014	201,463		
Stillbirth	Num/denom	1,818/496,293	1,605/451,727	0.97 (0.91, 1.04) *p =* 0.44	0.95 (0.89, 1.02) *p =* 0.16	477/136,161	481/126,093	1.09 (0.96, 1.24) *p =* 0.18	1.08 (0.95, 1.23) *p =* 0.24	845/224,014	736/201,463	0.97 (0.88, 1.07) *p =* 0.58	0.93 (0.84, 1.03) *p =* 0.18
	Percent	0.37	0.36			0.35	0.38			0.38	0.37		
Preterm birth	Num/denom	29,983/492,076	25,633/429,408	0.98 (0.96, 1.00) *p =* 0.02	0.96 (0.94, 0.97) *p <* 0.001	8,246/134,782	7,403/124,907	0.97 (0.94, 1.00) *p =* 0.05	0.95 (0.92, 0.99) *p =* 0.01	13,649/22,214	11,082/181,609	0.99 (0.97, 1.02) *p =* 0.62	0.97 (0.94, 0.99) *p =* 0.01
	Percent	6.1	6.0			6.1	5.9			6.1	6.1		
Small for gestational age	Num/denom	28,514/489,054	23,681/426,497	0.95 (0.94, 0.97) *p <* 0.001	0.95 (0.93, 0.96) *p <* 0.001	7,701/133,949	7,210/123,966	1.01 (0.98, 1.08) *p =* 0.45	1.01 (0.98, 1.04) *p =* 0.58	13,007/220,811	9,552/180,496	0.91 (0.88, 0.93) *p <* 0.001	0.90 (0.87, 0.92) *p <* 0.001
	Percent	5.8	5.6			5.8	5.8			5.9	5.3		
Induction of labour**	Num/denom	135,937/348,007	125,176/310,135	1.04 (1.03, 1.05) *p <* 0.001	1.04 (1.03, 1.05) *p <* 0.001	37,468/95,180	34,348/87,225	1.00 (0.98, 1.02) *p =* 0.82	0.99 (0.97, 1.01) *p =* 0.24	61,461/156,842	55,800/136,880	1.05 (1.04, 1.07) *p <* 0.001	1.05 (1.03, 1.06) *p <* 0.001
	Percent	39.1	40.4			39.4	39.4			39.2	40.8		
Elective cesarean section	Num/denom	64,079/496,293	62,552/451,727	1.08 (1.07, 1.09) *p <* 0.001	1.13 (1.11, 1.14) *p <* 0.001	17,020/136,161	17,045/126,093	1.09 (1.07, 1.12) *p <* 0.001	1.08 (1.06, 1.11) *p <* 0.001	29,529/224,014	28,539/201,463	1.08 (1.07, 1.10) *p <* 0.001	1.19 (1.17, 1.21) *p <* 0.001
	Percent	12.9	13.9			12.5	13.5			13.2	14.2		
Emergency cesarean section	Num/denom	84,156/496,293	82,975/451,727	1.10 (1.09, 1.11) *p <* 0.001	1.07 (1.06, 1.08) *p <* 0.001	22,974/136,161	21,841/126,093	1.03 (1.01, 1.05) *p =* 0.01	1.00 (0.98, 1.02) *p =* 0.95	38,595/224,014	38,623/201,463	1.14 (1.12, 1.16) *p <* 0.001	1.11 (1.09, 1.12) *p <* 0.001
	Percent	17.0	18.4			16.9	17.3			17.2	19.2		
Instrumental birth	Num/denom	61,256/496,293	57,602/451,727	1.04 (1.02, 1.05) *p <* 0.001	1.01 (1.00, 1.02) *p =* 0.16	16,427/136,161	15,975/126,093	1.06 (1.03, 1.08) *p <* 0.001	1.04 (1.01, 1.06) *p =* 0.002	27,807/224,014	25,733/201,463	1.03 (1.01, 1.05) *p =* 0.002	0.99 (0.97, 1.01) *p =* 0.40
	Percent	12.3	12.8			12.1	12.7			12.4	12.9		
Unassisted birth	Num/denom	285,525/496,293	247,474/451,727	0.90 (0.89, 0.90) *p <* 0.001	0.90 (0.89, 0.91) *p <* 0.001	79,416/136,161	70,914/126,093	0.92 (0.91, 0.93) *p <* 0.001	0.95 (0.93, 0.96) *p <* 0.001	127,517/224,014	108,091/201,463	0.88 (0.87, 0.89) *p <* 0.001	0.87 (0.86, 0.88) *p <* 0.001
	Percent	57.5	54.8			58.3	56.2			56.9	53.7		
Prolonged maternal length of stay	Num/denom	96,177/481,458	73,047/437,573	0.80 (0.79, 0.81) *p <* 0.001	0.77 (0.76, 0.78) *p <* 0.001	26,199/132,143	18,742/122,872	0.73 (0.71, 0.74) *p <* 0.001	0.70 (0.68, 0.71) *p <* 0.001	44,299/217,319	34,165/194,298	0.83 (0.82, 0.85) *p <* 0.001	0.80 (0.78, 0.81) *p <* 0.001
	Percent	20.0	16.7			19.8	15.3			20.4	17.6		
Maternal readmission**	Num/denom	15,888/418,393	12,725/431,164	0.89 (0.87, 0.91) *p <* 0.001	0.88 (0.86, 0.90) *p <* 0.001	4,387/132,120	3,161/122,851	0.77 (0.73, 0.81) *p <* 0.001	0.75 (0.72, 0.79) *p <* 0.001	7,172/217,289	5,802/187,920	0.93 (0.90, 0.97) *p <* 0.001	0.91 (0.88, 0.95) *p <* 0.001
	Percent	3.3	3.0			3.3	2.6			3.3	3.1		

aOR, adjusted odds ratio; CI, confidence interval; Num/denom, numerator/denominator.

*aORs from multi-level logistic regression models adjusted for maternal age, obstetric history, and comorbidities. All missing values for maternal characteristics and outcomes were imputed. *p*-Value from *t* test for the null hypothesis that the OR is equal to 1.

**Induction of labour denominator is restricted to women who did not have an elective cesarean section; maternal readmission denominator is restricted to women who gave birth up to and including 17 February 2021 and were discharged within 42 days of delivery.

Compared with corresponding pre-pandemic figures (Table 2), there was a small reduction in preterm birth rates during the entire pandemic period (6.0% versus 6.1% pre-pandemic, aOR 0.96, 95% CI 0.94 to 0.97, *p <* 0.001), the first lockdown period (5.9% versus 6.1% pre-pandemic, aOR 0.95, 95% CI 0.92 to 0.99, *p =* 0.01), and the second lockdown period (6.1% versus 6.1% pre-pandemic, aOR 0.97, 95% CI 0.94 to 0.99, *p =* 0.01).

Compared with the corresponding pre-pandemic periods, SGA rates were lower for the entire pandemic period (5.6% versus 5.8% pre-pandemic, aOR 0.95, 95% CI 0.93 to 0.96, *p <* 0.001) and for the second lockdown period (Fig 1; Table 2; 5.3% versus 5.9% pre-pandemic, aOR 0.90, 95% CI 0.87 to 0.92, *p <* 0.001), but there was no statistically significant difference during the first lockdown period (5.8% versus 5.8% pre-pandemic, aOR 1.01, 95% CI 0.98 to 1.04, *p =* 0.58).

Induction of labour rates were higher during the pandemic period overall (40.4% versus 39.1% pre-pandemic, aOR 1.04, 95% CI 1.03 to 1.05, *p <* 0.001) and in the second lockdown period (40.8% versus 39.2%, aOR 1.05, 95% CI 1.03 to 1.06, *p <* 0.001), but not in the first lockdown period (39.4% in both periods, aOR 0.99, 95% CI 0.97 to 1.01, *p =* 0.24) (Fig 1; Table 2). Rates of elective and emergency cesarean section were higher overall in the pandemic period (aOR 1.13, 95% CI 1.11 to 1.14, and aOR 1.07, 95% CI 1.06 to 1.08, respectively; both *p <* 0.001), primarily driven by the higher rates in the second lockdown period (aOR 1.19, 95% CI 1.17 to 1.21, and aOR 1.11, 95% CI 1.09 to 1.12, respectively; both *p <* 0.001). Instrumental births during the pandemic periods remained at similar levels to the corresponding pre-pandemic periods (Fig 1; Table 2).

The largest differences were seen for prolonged maternal length of stay (15.3% in the first lockdown versus 19.8% pre-pandemic, aOR 0.70, 95% CI 0.68 to 0.71, *p <* 0.001; 17.6% in the second lockdown versus 20.4% pre-pandemic, aOR 0.83, 95% CI 0.82 to 0.85, *p <* 0.001) and maternal readmission (2.6% in the first lockdown versus 3.3% pre-pandemic, aOR 0.77, 95% CI 0.73 to 0.81, *p <* 0.001; 3.1% in the second lockdown versus 3.3% pre-pandemic, aOR 0.91, 95% CI 0.88 to 0.95, *p <* 0.001) during the first and second lockdown periods compared to the corresponding pre-pandemic calendar periods. During the entire pandemic period, the rates of prolonged maternal length of stay and readmission were also significantly lower than pre-pandemic rates (Fig 1; Table 2).

There was evidence that the differences in rates varied according to women’s ethnic background for preterm births, cesarean births, and unassisted births (S3 Table), although this variation according to ethnic background was always small. The preterm birth rate was lower for white women during the pandemic period (6.1% pre-pandemic versus 5.9% during pandemic), but increased slightly for women of ethnic minority backgrounds (6.2% pre-pandemic to 6.3% during pandemic, *p-*value for interaction = 0.03). There was a greater increase in the rate of emergency cesarean birth for women of ethnic minority backgrounds (19.7% pre-pandemic to 21.9% during pandemic) compared to white women (15.9% pre-pandemic to 17.1% during pandemic, *p-*value for interaction = 0.02). There was a greater reduction in unassisted births for women of ethnic minority backgrounds than for white women (*p-*value for interaction < 0.001). Otherwise, the differences were similar in women from white and ethnic minority backgrounds.

There was no evidence that the differences in intervention rates and pregnancy outcomes varied according to socioeconomic background (all *p-*values for interaction > 0.05; S4 Table).

## Discussion

In this national study, we found that, overall, pregnancy outcomes during the COVID-19 pandemic were similar to outcomes in the corresponding calendar period 1 year earlier. There were small decreases in preterm birth and SGA birth rates and small increases in induction of labour and elective and emergency cesarean section rates during the pandemic period. Furthermore, there were lower rates of prolonged maternal length of stay and 42-day readmission, especially during the first lockdown period. Lastly, there was some evidence of a slightly different pattern of results in women from ethnic minority backgrounds, with a small increase in existing differences in the rates of preterm birth, emergency cesarean section, and unassisted vaginal delivery.

Our study concurs with preliminary reports from the ONS that rates of stillbirth in England were unchanged during the COVID-19 pandemic [10]. Moreover, we observed a lower number of singleton births overall during the pandemic compared to the pre-pandemic period, a phenomenon also noted by the ONS. However, unlike the ONS, which uses national birth registration data, our study found a statistically significant reduction in the rates of preterm birth and babies born SGA.

These observed differences between population-level analyses of the same population may partly be attributable to our analysis being restricted to singleton births. While multiple births represent a minority of all births (1.4%) [25], over half of multiple pregnancies result in birth before 37 weeks’ gestation [26]. In addition, it cannot be excluded that those undergoing ‘selective fertility’ (e.g., women requiring assisted conception), were less likely to become pregnant during these early months of the pandemic periods, giving birth in the later pandemic periods that were not covered by earlier studies in the UK [27].

This study considers interventions and outcomes at the time of birth that may have been affected by circumstances at different points throughout pregnancy. For example, fetal growth (measured as the proportion of babies born SGA) will reflect not only the impact of COVID-19 restrictions and clinical care experienced during the entire antenatal period, but also guidance by national maternity initiatives to reduce preterm birth and stillbirth [28–31]. In addition, there is a pre-existing national trend towards an increase in induction of labour, and this change in practice may have affected the results that we report [32]. Unfortunately, the dataset used does not contain information about antenatal care, and therefore it is not possible to directly attribute changes in outcomes to changes that have occurred as a result of the pandemic.

The reduction in preterm birth rate during the pandemic period was also demonstrated in an earlier UK-based study reporting neonatal admission rates [33]. Possible mechanisms for the observed reduction in preterm birth include modified population behaviour, resulting in reduced exposure to other pathogens including influenza, lower levels of physical exercise, and reduced workplace stress [34,35]. Similar findings have been observed in the preterm birth rate in China, with the rate returning to pre-pandemic levels after pandemic restrictions on societal interactions were lifted [36,37]. Changes in clinical practice due to the pandemic, such as reductions in labour induction and cesarean birth at earlier gestational age—perhaps due to a reduction in the identification of fetal growth restriction or preeclampsia due to reduced in-person appointments—could have also contributed to lower rates of preterm births [33].

During the pandemic period, our study population experienced higher rates of interventions including induction of labour and elective and emergency cesarean birth. To the best of our knowledge, similar findings have not been reported by other studies [38–40]. Although it is difficult to unpick the complex relationship between all factors influencing maternity care during the COVID-19 pandemic, it is likely that our findings of increased interventions are partially attributable to the centralisation of maternity services, including closure of midwife-led birth settings, which are associated with reduced interventions for women at low risk of complications [13,41,42]. Other potential explanations are that changes in care-seeking behaviour amongst pregnant women and an increase in virtual appointments may have created delays in the diagnosis of adverse conditions, which may have led in turn to missed opportunities for conservative management. Also, there may also have been a change in clinician behaviour towards a lower threshold for interventions in order to expedite births and to avoid emergency situations. Lastly, occupational stress and burnout among maternity staff could also be associated with a more interventionist approach [43].

Our results show a reduction in prolonged maternal hospital stays, a finding supported by other studies [44–46]; early discharge from the hospital may have been initiated by either the clinician or the woman to reduce the perceived risk of infection associated with hospital stay, or to reunite with family members unable to visit maternity units. Rates of maternal readmission were also reduced during the pandemic period, which may reflect that women were less likely to seek care for postpartum issues.

There is evidence from other clinical areas that people with ethnic minority backgrounds and those from more deprived communities were disproportionately affected by the COVID-19 pandemic [6,47,48]. Our results demonstrate little variation according to women’s ethnic background in the differences associated with the pandemic periods. This may be explained by recommendations from UK midwifery and obstetric professional bodies to be particularly attentive to women in these groups, given that they are considered to be at higher risk of complications from COVID-19 [12].

The main strengths of this study are its large size, with the study including almost all births in England over a period of almost 2 years, covering periods of high COVID-19 prevalence in the UK and capturing significant events along the pandemic timeline. HES data are well-established and have previously been used for national comparisons of maternity care [49]. The clinical and patient demographic information in HES (for both maternity and further inpatient episodes after birth) allowed for a broader range of maternity care and neonatal outcomes to be compared across COVID-19 periods than previous studies exploring the combined direct and indirect effects of the pandemic [7,39]. Access to individual data for each woman and infant, rather than national aggregate data, enabled comparison of differences in intervention and outcome rates according to women’s ethnic and socioeconomic backgrounds.

This study reports the differences in the rates of a number of interventions and outcomes between pandemic and pre-pandemic periods, also exploring whether these differences varied according to ethnic and socioeconomic background. These multiple comparisons, increasing the chance of false-positive results, should be taken into account, especially when interpreting small but statistically significant differences [50].

Our study raises questions about the potential associations between societal and care changes during the COVID-19 pandemic and maternal and perinatal outcomes. The pandemic can be regarded as a natural experiment in which many aspects of care and behaviour changed simultaneously. Our results suggest that these changes may have led to reductions in the rate of preterm and SGA birth as well as increases in obstetric intervention rates. All this requires further investigation, particularly as similar results have not, to the best of our knowledge, been seen in other high-income settings [7].

### Conclusion

In this study of all births in the English NHS, we found a small decrease in the rates of preterm and SGA birth during the pandemic, compared to the corresponding pre-pandemic calendar period 1 year earlier. There was a small increase in the rates of obstetric interventions, which may be a consequence of temporary closure of midwife-led birth settings or a response to delays in pregnant women seeking care. These results varied slightly according to women’s ethnic background, but not according to their socioeconomic background. All these findings should be interpreted with caution, because differences were small, many comparisons were made, and the relationships between the factors influencing maternity care and pregnancy outcomes during the COVID-19 pandemic are complex.

## Supporting information

S1 Fig Number of singleton births, per month. (TIF)

S1 Table Timeline of COVID-19 restrictions across England and the United Kingdom from 23 March 2020 to 22 February 2021. (DOC)

S2 Table Definitions of maternal characteristics and maternal and perinatal outcomes, and their coding and completeness in Hospital Episode Statistics (HES). Table A: Maternal characteristics. Table B: Maternal and perinatal outcomes.
(DOC)

S3 Table Comparisons of outcomes in the COVID-19 pandemic with outcomes in the same period in the previous year, by ethnic background. (DOC)

S4 Table Comparisons of outcomes in the COVID-19 pandemic with outcomes in the same period in the previous year, by deprivation. (DOC)

S1 Text RECORD statement—Checklist of items, extended from the STROBE statement, that should be reported in observational studies using routinely collected health data. (DOC)

## Abbreviations

aOR: adjusted odds ratio
HES: Hospital Episode Statistics
ICD-10: International Classification of Diseases–10th revision
IMD: Index of Multiple Deprivation
NHS: National Health Service
ONS: Office for National Statistics
OPCS-4: Office for Population Censuses and Surveys Classification of Interventions and Procedures–4th revision
OR: odds ratio
SGA: small for gestational age
