# Supplementary figures and images for: Obstetric interventions and pregnancy outcomes during the COVID-19 pandemic in England: A nationwide cohort study
Source: PLoS Med. 2022 Jan 10;19(1):e1003884. doi: 10.1371/journal.pmed.1003884 (PMC8803187; doi:10.1371/journal.pmed.1003884)

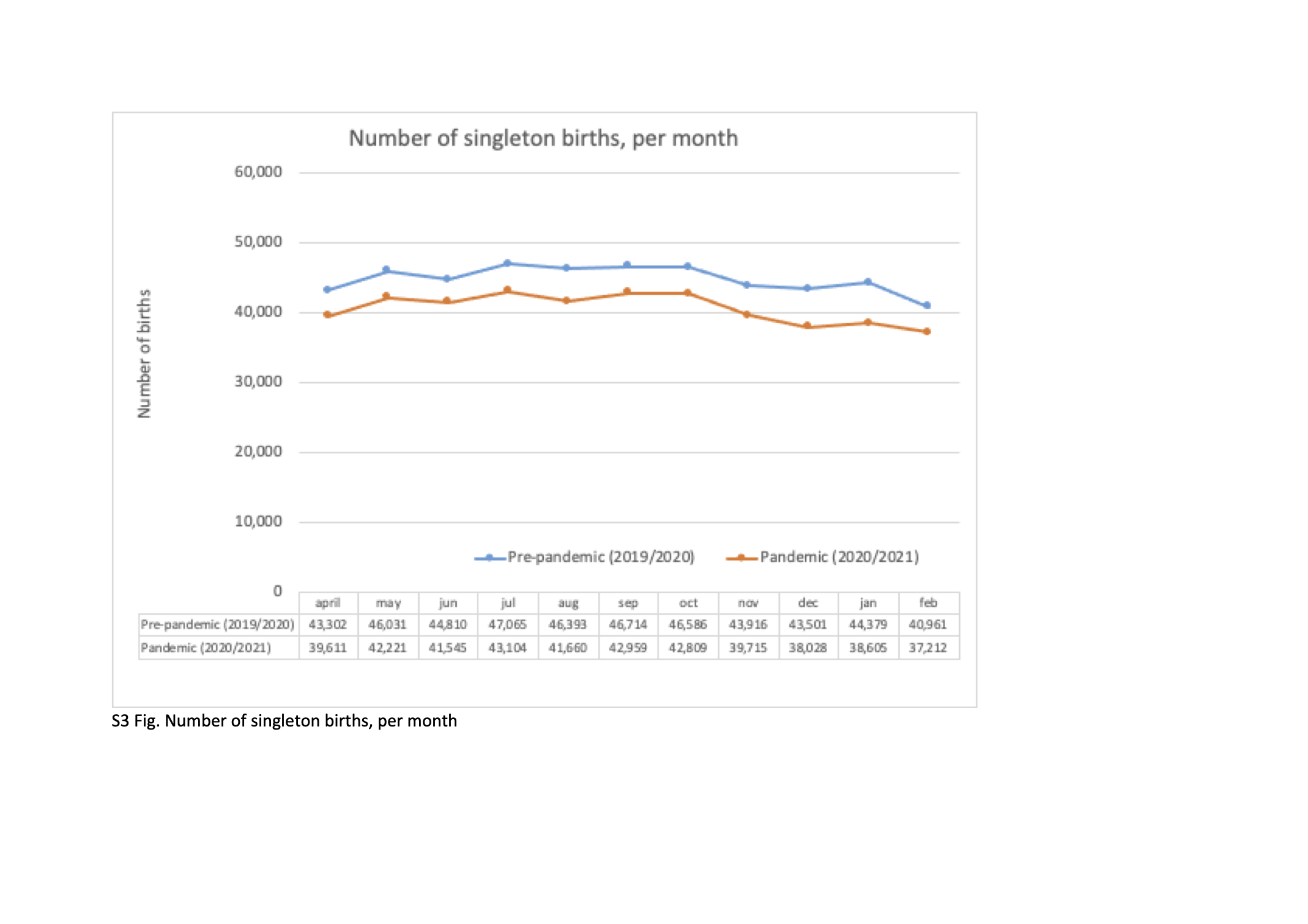

Supplement: S1 Fig — (TIF) [file pmed.1003884.s001.tif]
