# Supplementary material for: Obstetric interventions and pregnancy outcomes during the COVID-19 pandemic in England: A nationwide cohort study
Source: PLoS Med. 2022 Jan 10;19(1):e1003884. doi: 10.1371/journal.pmed.1003884 (PMC8803187; doi:10.1371/journal.pmed.1003884)
Supplement: S1 Table — (DOC) [file pmed.1003884.s002.doc]

| **Date** | **Nation/Region** | **Description** |
| --- | --- | --- |
| **COVID first lockdown period**  **23 March-23 June 2020** |  |  |
| 23 March 2020 | UK national lockdown announced | All non-essential high street businesses close and people ordered to stay at home, permitted to leave for essential purposes only – food, one hour of exercise per day. Only leave the house for work if unable to work from home. |
| 10 May 2020 | England | People permitted to leave home for unlimited outdoor recreation.  Return to work if unable to work from home. |
| 27 May 2020 | England | Launch of ‘test and trace’ system. |
| 1 June 2020 | England | Restrictions on leaving home replaced with requirement to be home overnight.  Meeting outside of up to six people permitted.  Year one and year six pupils return to school (age 4/5 and 10/11 years). |
| 15 June 2020 | England | All retail businesses permitted to open.  People who live alone or single parent families may form support bubble with one other household.  Year ten and twelve pupils return (age 14/15 and 17/18).  Public required to wear face coverings on public transport. |
| **COVID local restrictions**  **24 June – 21 September 2020** |  |  |
| 24 June | England | Announced that pubs, restaurants tourist attractions, some leisure facilities and hairdressers may reopen from 4 July.  Two households able to meet indoors.  Weddings permitted with max 30 attendees.  Holidays within England permitted. |
| 26 June 2020 | England | Quarantine regulations changed to allow for holidays to places such as Spain and Greece. |
| 27 June 2020 | UK | NHS calls for pregnant women from Black, Asian and Minority Ethnic groups to receive extra coronavirus checks and support due to vulnerability. |
| 1 July 2020 | Leicester | Local lockdown. |
| 3 July 2020 | England | List of 50 ‘reduced risk’ countries announced which require no quarantine on arrival from. |
| 25 July 2020 | England | All but essential travel to Spain advised against and 14-day quarantine reintroduced. |
| 30 July 2020 | North of England | Local lockdown. |
| 31 July 2020 | England | Reversal of decision to further ease lockdown postponing the reopening of some venues (such as casinos, bowling alleys) for two weeks. |
| 3 August | UK | Launch of ‘eat out to help out’ incentive scheme, aimed at encouraging the public to visit participating restaurants. |
| 15 August 2020 | England | Reopening of indoor play centres; indoor performances and wedding receptions allowed. |
| 9 September 2020 | England | Gatherings of more than six people banned. |
| **COVID second lockdown period**  **22 September 2020 – 22 February 2021** |  |  |
| 22 September 2020 | England | Return to working from home. |
| 14 October | England, regional fluctuations from tiers 2-3. | Three tier system, 1 = medium, 2 = high, 3 = very high. |
| 31 October | England | Full national lockdown announced to come into force on 5 November. |
| 5 November | England | Full National lockdown. Non-essential retail, pubs, restaurants close. University, schools and courts remain open. |
| 2 December | England | Return to three tier system. |
| 19 December | South East England | New strain identified. |
|  | Scotland | Announces travel ban with the rest of the UK. |
| 21 December | London and South-East England | Tier 4 comes into force. |
| 25 December | England | Rules relaxed for tiers 1-3, three households allowed to meet for Christmas Day.  Tier 4, no relaxation of rules, certain exceptions included support bubbles. |
| 26 December 2020 | England | Return to tiered restrictions, with more regions moving to tier 4. |
| 4 January 2021 | England | Lockdown announced from 6 January including school closures. |
| 27 January 2021 | England | Compulsory hotel quarantine announced for those arriving from high-risk countries. |
| 22 February 2021 | England | Four-step plan for easing restrictions announced, with schools reopening and outdoor socialising with one other person allowed from 8 March 2021. |

**S1 Table: Timeline of COVID-19 restrictions across England and the United Kingdom from 23 March 2020 to 22 February 2021^1,2,3^**

References

1. British Foreign Policy Group. COVID-19 Timeline. 2021 [cited 10 Oct 2021]. Available: https://bfpg.co.uk/2020/04/covid-19-timeline/
2. Institute for Government analysis. Timeline of UK coronavirus lockdowns, March 2020 to March 2021. 2021 [cited 12 Mar 2021]. Available: <https://www.instituteforgovernment.org.uk/sites/default/files/timeline-lockdown-web.pdf>
3. Brown J, Kirk-Wade E. Coronavirus: A history of English lockdown laws. London; 2021. Available: https://researchbriefings.files.parliament.uk/documents/CBP-9068/CBP-9068.pdf
