# Supplementary material for: Obstetric interventions and pregnancy outcomes during the COVID-19 pandemic in England: A nationwide cohort study
Source: PLoS Med. 2022 Jan 10;19(1):e1003884. doi: 10.1371/journal.pmed.1003884 (PMC8803187; doi:10.1371/journal.pmed.1003884)
Supplement: S2 Table — Table A: Maternal characteristics. Table B: Maternal and perinatal outcomes. (DOC) [file pmed.1003884.s003.doc]

| **Maternal characteristics** | **Coding** | **Missing values** |
| --- | --- | --- |
| Maternal age | Defined using startage field in HES; grouped into five categories:  Less than 20, 20-24, 25-29, 30-34, 35-39, 40 and over | *9 / 948,020 (0.001%)* |
| Obstetric history | Defined using numpreg variable in HES, and women’s previous HES hospital admission records: grouped into three categories: primiparous, multiparous without previous CS, multiparous with previous CS | *None* |
| Pre-existing diabetes | Defined using ICD10 codes E10, E11, E13, E14, O24.0, O24.1, O24.2, O24.3 | *None* |
| Gestational diabetes | Defined using ICD10 code O24.4 | *None* |
| Pre-existing hypertension | Defined using ICD10 codes I10-I15, O10, O11 | *None* |
| Pre/eclampsia | Defined using ICD10 codes O14, O15 | *None* |
| COVID19 status at birth | Defined using ICD10 code U07.1 | *None* |
| Maternal ethnicity | Defined using ethnos variable in HES; grouped into four categories: white, South Asian, Black and Other | *106,583 / 948,020 (11.6%)* |
| Maternal deprivation | Defined using imd19rk variable in HES; grouped into five quintiles | *6381 / 948,020 (0.7%)* |

**S2 Table A: Definitions of maternal characteristics, their coding and completeness in Hospital Episode Statistics (HES)**

| **Outcome** | **Numerator / coding** | **Denominator / coding** | *Missing values* |
| --- | --- | --- | --- |
| Stillbirth | Defined using ICD10 code (Z37.1) OR birth status field (birstat_1=2,3,4) in maternity tail for providers with over 95% data completeness.  * In the UK, stillbirth is defined as birth without signs of life occurring at or after 24+0 completed gestational weeks based on estimated due date calculated using universally defined ultrasound scan at 11-13 weeks’ gestation. | All singleton births | *None* |
| Preterm birth | Defined using gestational age field in HES maternity tail | All singleton births, excluding records missing information on gestational age | *23,536 / 948,020 (2.5%)* |
| Small-for-gestational-age | Defined as less than the 10th birthweight centile using the WHO-UK 1990 charts. Birthweight centiles are calculated using birthweight, gestational age, sex of baby fields in maternity tail. | All singleton births, excluding records missing information on gestational age, birthweight or sex of baby | *29,469 / 948,020*  *(3.1%)* |
| Induction of labour | Defined using the delivery onset field (delonset=3,4,5) from the maternity tail. Failed induction (ICD-10 code O61) is also included in the numerator as this represents intention to treat. | All singleton births, excluding elective caesarean section; and records missing information on delivery onset | *163,247 / 821,389*  *(19.9%)* |
| Elective caesarean section | Defined using OPCS code R17 | All singleton births | *None* |
| Emergency caesarean section | Defined using OPCS codes R18/R25.1 | All singleton births | *None* |
| Instrumental birth | Defined using OPCS codes R21/R22 | All singleton births | *None* |
| Unassisted birth | Defined using OPCS code R23/R24 | All singleton births | *None* |
| Length of stay post birth  (3 or more days) | Defined as the number of days between date of discharge and date of admission for the birth episode. | All singleton births with non-missing date of discharge information | *25,989 / 944,565*  *(2.8%)* |
| Maternal readmission  (42-days) | Defined as unplanned, overnight readmission to hospital within 42 days of giving birth, excluding those accompanying an unwell baby. Mothers readmitted `with the following admission method codes: 21, 22, 23, 24, 28, 2A, 2B, 2D, 31, 32, 82, 83 within 42 days of birth. | All singleton births with non-missing date of discharge information and date of delivery before 17 February 2021 (to allow for six-week follow up). Women who died before discharge or were not discharged within 42 days of delivery were excluded. | *None* |

**S2 Table B: Definitions of maternal and perinatal outcomes, their coding and completeness in Hospital Episode Statistics (HES)**
