# Supplementary material for: Obstetric interventions and pregnancy outcomes during the COVID-19 pandemic in England: A nationwide cohort study
Source: PLoS Med. 2022 Jan 10;19(1):e1003884. doi: 10.1371/journal.pmed.1003884 (PMC8803187; doi:10.1371/journal.pmed.1003884)
Supplement: S4 Table — (DOC) [file pmed.1003884.s005.doc]

|  | **Pre-pandemic vs Pandemic comparison** | | | | | | | | | |
| --- | --- | --- | --- | --- | --- | --- | --- | --- | --- | --- |
|  | Time period covered: 23 March - 22 February | | | | | | | | | |
|  | **Less deprived** | | **More deprived** | | **Impact of pandemic** | | **Impact of deprivation** | | **Interaction term** | |
|  | **Pre-pandemic** | **Pandemic** | **Pre-pandemic** | **Pandemic** | **OR**  **(95% CI)**  **p-value** | **aOR***  **(95% CI)**  **p-value** | **OR**  **(95% CI)**  **p-value** | **aOR***  **(95% CI)**  **p-value** | **OR**  **(95% CI)**  **p-value** | **aOR***  **(95% CI)**  **p-value** |
|  | **num/denom**  **(%)** | **num/denom**  **(%)** | **num/denom**  **(%)** | **num/denom**  **(%)** |  |  |  |  |  |  |
| Stillbirth | 761/252226 | 641/232476 | 1041/240322 | 951/216615 | 0.92  (0.83,1.02)  p=0.12 | 0.90 (0.81,1.00)  p=0.05 | 1.40 (1.27,1.54)  p<0.001 | 1.40 (1.27,1.55)  p<0.001 | 1.11 (0.96,1.27)  p=0.15 | 1.10 (0.96,1.27)  p=0.16 |
|  | 0.30 | 0.28 | 0.43 | 0.44 |  |  |  |  |  |  |
| Preterm birth | 13381/250027 | 11508/220677 | 16393/238330 | 13986/206262 | 0.97  (0.95,1.00)  p=0.03 | 0.95 (0.93,0.97)  p<0.001 | 1.28 (1.24,1.31)  p<0.001 | 1.25 (1.20,1.27)  p<0.001 | 1.01 (0.98,1.05)  p=0.41 | 1.01 (0.98,1.05)  p=0.52 |
|  | 5.4 | 5.2 | 6.9 | 6.8 |  |  |  |  |  |  |
| Small-for-gestational-age | 12167/248699 | 10317/219415 | 16059/236650 | 13194/204621 | 0.96 (0.94,0.99)  p=0.004 | 0.96 (0.93,0.98)  p<0.001 | 1.38 (1.35,1.42)  p<0.001 | 1.40 (1.37,1.44)  p<0.001 | 0.99 (0.95,1.02)  p=0.46 | 0.99 (0.95,1.02)  p=0.41 |
|  | 4.9 | 4.7 | 6.8 | 6.4 |  |  |  |  |  |  |
| Induction of labour** | 65624/173006 | 61844/157684 | 69267/172191 | 62602/150706 | 1.02 (1.01,1.04) p=0.001 | 1.04 (1.02,1.05) p<0.001 | 1.12 (1.11,1.14)  p<0.001 | 1.07 (1.05,1.08)  p<0.001 | 1.00 (0.99,1.02)  p=0.76 | 1.00 (0.98,1.02)  p=0.79 |
|  | 37.9 | 39.2 | 40.2 | 41.5 |  |  |  |  |  |  |
| Elective cesarean section | 35184/252226 | 34468/232476 | 28471/240322 | 27718/216615 | 1.07 (1.06,1.09)  p<0.001 | 1.12 (1.10,1.14)  p<0.001 | 0.83 (0.82,0.85)  p<0.001 | 0.85 (0.84,0.87)  p<0.001 | 1.02 (0.99,1.04)  p=0.17 | 1.02 (0.99,1.05)  p=0.13 |
|  | 13.9 | 14.8 | 11.8 | 12.8 |  |  |  |  |  |  |
| Emergency cesarean section | 42112/252226 | 41834/232476 | 41317/240322 | 40557/216615 | 1.09 (1.08,1.11)  p<0.001 | 1.07 (1.06,1.09)  p<0.001 | 1.03  (1.02,1.05)  p<0.001 | 1.10 (1.08,1.12)  p<0.001 | 1.01  (0.99,1.03)  p=0.27 | 1.00 (0.98,1.02)  p=0.82 |
|  | 16.7 | 18.0 | 17.2 | 18.7 |  |  |  |  |  |  |
| Instrumental birth | 33885/252226 | 32025/232476 | 26841/240322 | 25199/216615 | 1.03 (1.01,1.053)  p=0.001 | 1.01 (0.99,1.03)  p=0.34 | 0.81 (0.80,0.83)  p<0.001 | 0.87 (0.86,0.89)  p<0.001 | 1.02 (0.99,1.04)  p=0.21 | 1.00 (0.98,1.03)  p=0.90 |
|  | 13.4 | 13.8 | 11.2 | 11.6 |  |  |  |  |  |  |
| Unassisted birth | 140426/252226 | 123649/232476 | 143052/240322 | 122521/216615 | 0.91 (0.90,0.92)  p<0.001 | 0.90 (0.89,0.92)  p<0.001 | 1.17 (1.15,1.18)  p<0.001 | 1.08 (1.06,1.09)  p<0.001 | 0.98 (0.96,1.00)  p=0.02 | 0.99 (0.98,1.01)  p=0.46 |
|  | 55.7 | 53.2 | 59.5 | 56.6 |  |  |  |  |  |  |
| Maternal length of stay | 46572/244217 | 35650/224763 | 48835/233599 | 36879/210281 | 0.80 (0.79,0.81)  p<0.001 | 0.77 (0.76,0.79)  p<0.001 | 1.14 (1.12,1.16)  p<0.001 | 1.17 (1.16,1.19)  p<0.001 | 1.01 (0.99,1.03)  p=0.56 | 0.99 (0.97,1.01)  p=0.52 |
|  | 19.1 | 15.9 | 20.9 | 17.5 |  |  |  |  |  |  |
| Maternal readmission** | 8379/244189 | 6669/221436 | 7393/233562 | 5967/207244 | 0.88 (0.85,0.90)  p<0.001 | 0.86 (0.84,0.89)  p<0.001 | 0.96 (0.93,1.00)  p=0.03 | 0.99 (0.96,1.02)  p=0.55 | 1.04 (0.99,1.09)  p=0.10 | 1.03 (0.99,1.08)  p=0.17 |
|  | 3.4 | 3.0 | 3.2 | 2.9 |  |  |  |  |  |  |
| **aOR, adjusted odds ratio, CI confidence interval from multi-level logistic regression models, adjusted for maternal age, obstetric history and comorbidities. All missing values for maternal characteristics and outcomes were imputed. P-value from t-test for the null hypothesis that the OR is equal to 1. **Induction of labour denominator is restricted to women who did not have an elective cesarean section, maternal readmission denominator is restricted to women who gave birth before 17 February 2021 and were discharged within 42 days of delivery.* | | | | | | | | | | |

**S4 Table. Comparisons of outcomes in the COVID-19 pandemic with the same period in the previous year, by deprivation**
